# Multielemental analysis of 20 mushroom species growing near a heavily trafficked road in Poland

**DOI:** 10.1007/s11356-016-6760-8

**Published:** 2016-05-07

**Authors:** M. Mleczek, P. Niedzielski, P. Kalač, A. Budka, M. Siwulski, M. Gąsecka, P. Rzymski, Z. Magdziak, K. Sobieralski

**Affiliations:** 1 Department of Chemistry, Poznań University of Life Sciences, Poznań, Poland; 2 Faculty of Chemistry, Adam Mickiewicz University in Poznań, Poznań, Poland; 3 Department of Applied Chemistry, Faculty of Agriculture, University of South Bohemia, České Budějovice, Czech Republic; 4 Department of Mathematical and Statistical Methods, Poznań University of Life Sciences, Poznań, Poland; 5 Department of Vegetable Crops, Poznań University of Life Sciences, Poznań, Poland; 6 Department of Environmental Medicine, Poznań University of Medical Sciences, Poznań, Poland

**Keywords:** Contamination, Consumer health, Trace elements, Environment, Mushrooms

## Abstract

The aim of this work was to compare 10 mostly edible aboveground and 10 wood-growing mushroom species collected near a heavily trafficked road (approximately 28,000 vehicles per 24 h) in Poland with regard to their capacity to accumulate 26 trace elements (Ag, Al, As, Au, B, Ba, Bi, Cd, Co, Cr, Cu, Fe, Ga, Ge, In, Li, Mn, Ni, Pb, Re, Sb, Se, Sr, Te, Tl, and Zn) in their fruit bodies in order to illustrate mushroom diversity in element accumulation. All analyses were performed using an inductively coupled plasma optical emission spectrometry (ICP-OES) spectrometer in synchronous dual view mode. The aboveground species had significantly higher levels of 12 elements, including Ag, As, Pb, and Se, compared to the wood-growing species. An opposite relationship was observed only for Au, Ba, and Sr. The results of principal component analysis (PCA) and hierarchical cluster analysis (HCA) implied some new relationships among the analyzed species and elements. Of the analyzed mushroom species, lead content in *Macrolepiota procera* would seem to pose a health risk; however, at present knowledge regarding lead bioaccessibility from mushrooms is quite limited.

**Electronic supplementary material:**

The online version of this article (doi:10.1007/s11356-016-6760-8) contains supplementary material, which is available to authorized users.

## Introduction

Mushroom consumption is generally high in Eastern Europe and Asia. However, consumption of wild mushrooms collected from polluted areas may be associated with the risk of exposure to toxic elements (particularly Cd, Hg, Pb, and As) being introduced to the human body. The content of trace elements in wild growing mushroom species, both edible and inedible, has been reported in numerous articles (for an overview of data, see reviews of Kalač and Svoboda (2000), Kalač (2010), and Falandysz and Borovička (2013)) and some recent papers, e.g., Kaya and Bag (2010), Ayaz et al. (2011), Sarikurkcu et al. (2015), Yin et al. (2012), or Mleczek et al. (2015a). Mushrooms were analyzed either as one species or as a comparison of many species, e.g., in a study of Borovička and Řanda (2007), where 130 species were compared. Additionally, wild growing mushroom species have been characterized on the basis of several elements, rarely on the content of selected forms (chemical species) only (e.g., Falandysz et al. 2007; Niedzielski et al. 2013) or based on a multielemental analysis (e.g., Gucia et al. 2012a; Kojta et al. 2012). Mushrooms have been collected from both unpolluted (e.g., Falandysz et al. 2012) and polluted areas (e.g., Svoboda et al. 2006; Radulescu et al. 2010; Árvay et al. 2014; Mleczek et al. 2015b).

Variability in the chemical composition of mushrooms within a species is greater than that of plants, much more so than within cultivars of a crop. Each individual fruit body can result from the cross-breeding of different hyphae and so presents a distinct genotype. Contents of a trace element in a mushroom species therefore vary widely, even by as much as 1 order of magnitude. Mushroom species, but most probably not genus, level of substrate composition, and local pollution with trace elements are the principal factors affecting trace element level in fruit bodies. The lifetime of most fruit bodies is usually only 10–14 days. Thus the role of fruit body age and size is of less importance owing to the limited proportion of contaminants originating from atmospheric depositions. However, this role may increase in some wood-growing species that have a longer lifespan than is usual in aboveground species (for detailed information, see reviews of Kalač and Svoboda (2000), Kalač (2010), and Falandysz and Borovička (2013)).

Mushrooms not only accumulate elements from underlying substrate but also from host trees depending on the specificity of species. Elements detrimental to humans originate from both natural sources and anthropogenic activities. Motor transport delivers significant amounts of both gaseous pollutants and selected elements, which generally negatively influence human health (Künzli et al. 2000) and contribute to changes in climate (EPA 2015). In Poland, apart from the sale of cultivated mushrooms in shops, wild growing mushroom species are frequently sold along roadsides and in street markets. Therefore, the aim of this study was to illustrate the diversity of 10 aboveground mushroom species and 10 wood-growing species, collected near a busy trunk road, in the accumulation 26 trace elements: silver (Ag), aluminum (Al), arsenic (As), gold (Au), boron (B), barium (Ba), bismuth (Bi), cadmium (Cd), cobalt (Co), chromium (Cr), copper (Cu), iron (Fe), gallium (Ga), germanium (Ge), indium (In), lithium (Li), manganese (Mn), nickel (Ni), lead (Pb), rhenium (Re), antimony (Sb), selenium (Se), strontium (Sr), tellurium (Te), thallium (Tl), and zinc (Zn). The determined content of these elements in the fruit bodies of the mushrooms also allowed an assessment to be made of the health risks associated with the consumption of mushrooms collected near frequented roads.

## Materials and methods

### Experimental material

**Fig. 1:**
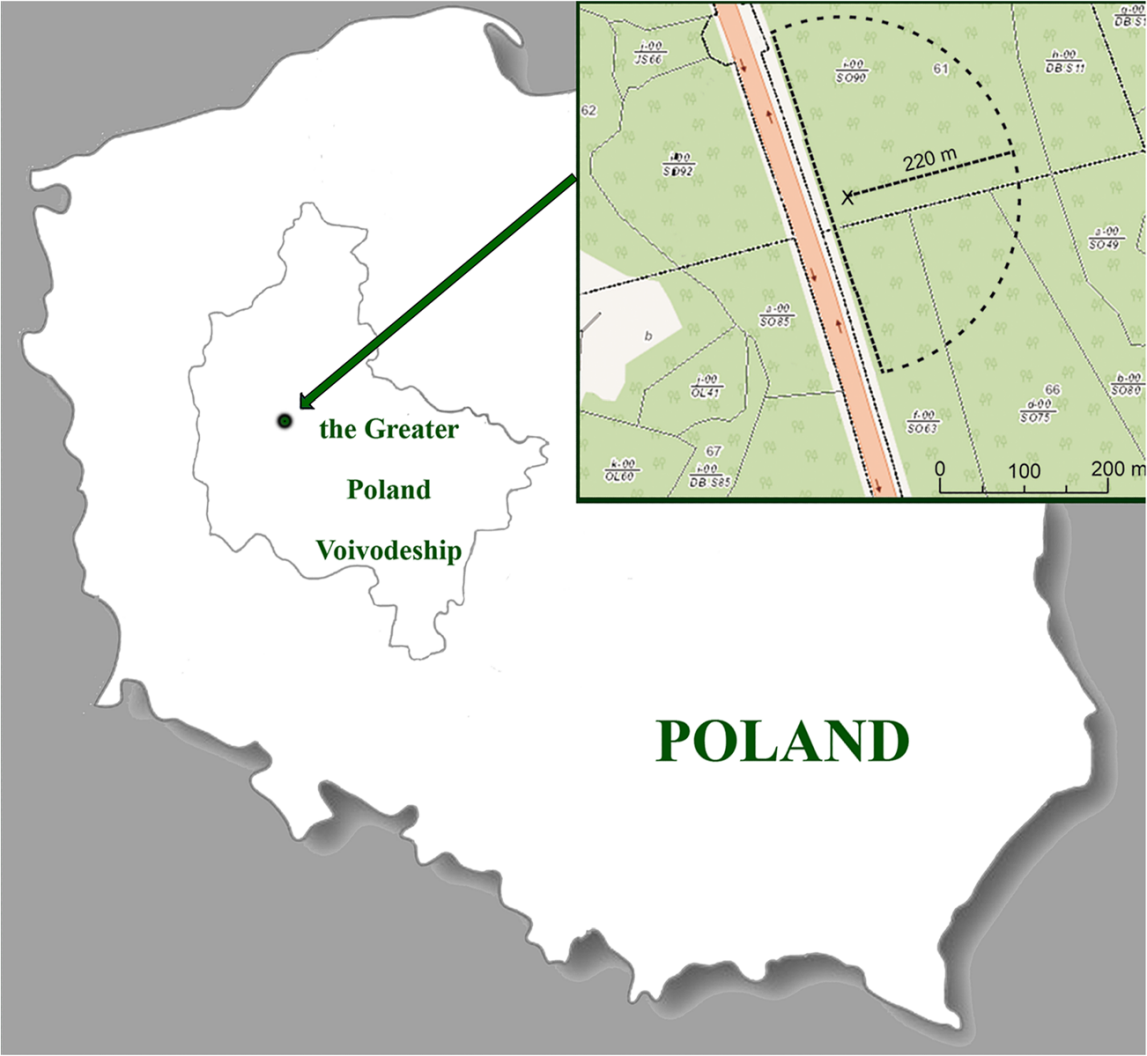
Map of the sampled area

This work continues the theme of our previous paper (Mleczek et al. 2016), in which we analyzed the content of Platinum Group Elements (PGEs) and Rare Earth Elements (REEs) in the same 20 mushroom species growing near (up to 40 m) the S11, a heavily trafficked road in Poland (about 28,000 vehicles per 24 h) (GDDKiA 2005, 2010). The sampled area lay up to 220 m from the localization point described by coordinates 52°16′50.75″N, 17°03′43.31″E (Fig. 1).

For 3 weeks prior to the day of sample collection, the prevailing direction of winds was south-east with a mean speed 1 m s^−1^. It is worth noting that the direction of local winds is generally variable. The mean temperature was 10 ± 1 °C, atmospheric pressure 994 ± 3 hPa, and relative humidity was 96 ± 1 %. According to data presented at the Voivodeship Environmental Protection Inspectorate in Poznań (http://powietrze.poznan.wios.gov.pl/), concentration of nitric oxide (NO), nitrogen dioxide (NO_2_), sulfur dioxide (SO_2_), and ozone (O_3_) was 1 ± 3, 21 ± 6, 1.1 ± 0.4, and 31 ± 13 μg m^−3^, respectively. The mean concentration of particulate matter (PM10—10 μm or less in diameter) was 16. All presented meteorological data were determined within 24 h of October 22, 2014, when the sampled, fully developed fruit bodies were collected at the same time. Mushroom species were collected from a mixed forest composed of four tree species: *Acer* L. (*Sapindaceae*), *Acacia* Mill. (*Fabaceae s.l.*), *Pinus* L. (*Pinaceae* Lindl.) being the main species, and *Quercus* L. (*Fagaceae* Dumort.). The main criteria for mushroom selection were their occurrence in a confined sampling area and proximity to a heavily trafficked road. This sampling location was selected from among six possible sites due to the greatest occurrence of mushroom fruit bodies being observed there over a period of 5 years. The altitude of experimental site ranged from 83 to 85 m above sea level.

Soils where mushrooms were collected were formed from outwash sands, and according to the Classification of Polish wood soils (Czępińska-Kamińska et al. 2000) they belong to rusty soils. The term has no direct equivalent in WRB international soil classification (IUSS Working Group WRB 2015).

The collected mushroom species were divided into two groups: aboveground mushroom species and wood-growing species (Table 1). The number of collected fruit bodies for each of the collected species is also given.

**Table 1:** Analyzed mushroom species and their edibility

Species	Number of samples	Edibility
Wood-growing species		
*Armillaria mellea* (Vahl) P. Kumm.	5	Edible
*Auricularia auricula-judae* (Bull.) Quél.	3	Edible
*Flammulina velutipes* (Curtis) Singer	3	Edible
*Ganoderma applanatum* (Pers.) Pat.	4	Inedible
*Grifola frondosa* (Dicks.) Gray	3	Edible
*Laetiporus sulphureus* (Bull.) Murrill	4	Inedible
*Piptoporus betulinus* (Bull.) P. Karst.	4	Inedible
*Pleurotus ostreatus* (Jacq.) P. Kumm.	5	Edible
*Pleurotus* spp.	8	Edible
*Polyporus squamosus* (Huds.) Fr.	4	Edible
Aboveground species		
*Cantharellus cibarius* Fr.	8	Edible
*Laccaria amethystina* (Huds.) Cooke	4	Edible
*Leccinum scabrum* (Bull.) Gray	5	Edible
*Lepista gilva* (Pers.) Pat.	6	Edible
*Lyophyllum fumosum* (Pers.) P.D. Orton	5	Edible
*Macrolepiota procera* (Scop.) Singer	3	Edible
*Paxillus involutus* (Batsch) Fr.	3	Inedible
*Suillus bovinus* (L.) Roussel	11	Edible
*Suillus luteus* (L.) Roussel	3	Edible
*Tricholoma equestre* (L.) P. Kumm.	3	Edible

After the transport to the laboratory, all the bodies were carefully cleaned with distilled water using Milli-Q Advantage A10Water Purification Systems, Merck Millipore (Merck, Darmstadt, Germany) to exclude any external surface contamination. The collected materials were dried in an electric drying oven (SLW 53 STD, Pol-Eko, Wodzisław Śląski, Poland) for 96 h at 65 ± 1 °C to a constant weight and powdered using a Cutting Boll Mill 200 (Retsch GmbH, Haan, Germany) for 2 min.

### Analytical methods

Accurately weighed samples of 0.300 ± 0.001 g were digested with concentrated Suprapure (TM) (65 %) nitric acid (Merck, Darmstadt, Germany) in closed teflon containers in a microwave digestion system Mars 5 (CEM, Matthews, NC, USA). The digest was then filtered through a paper filter (Qualitative Filter Papers, Grade 595: 4–7 μm (Whatman, Maidstone, UK)) and diluted to 15.0 mL with water (purified in an ion-exchange/reverse osmosis system (Millipore, Billerica, USA)).

All analyses were performed using an inductively coupled plasma optical emission spectrometry (ICP-OES) spectrometer (model 5100 Agilent, USA) in synchronous dual view (simultaneous axial and radial plasma observation) mode. Common conditions for the determination of 26 elements were as follows: RF power 1.2 kW, nebulizer gas flow 0.7 L min^−1^, auxiliary gas flow 1.0 L min^−1^, plasma gas flow 12.0 L min^−1^, CCD temperature −40 °C, viewing height for radial plasma observation 8 mm, accusation time 5 s, three replicates. Spectral conditions for optical emission detection are collected in Table S1 and S2 (Supplementary data).

Each of the individual fruit bodies was analyzed in triplicate. In order to consolidate the number of replications while maintaining randomization, a triple sampling with replacement from the pool of individuals found for each species was conducted. All trace element contents are given as milligram per kilogram dry matter (d.m.).

### Analytical quality control (QC) and quality assurance (QA)

The detection limits were determined as 3-sigma criteria at the level of 0.01 mg kg^−1^ d.m. for all the elements determined. The correlation coefficients for all calibration curves were higher than 0.9995, the uncertainty level for the whole analytical process (including sample preparation) was 20 %. The traceability was checked using five reference materials (biological and geological); the results of the analysis of certified reference materials are included in Table S3 (Supplementary data). The recovery was acceptable for all the elements determined. For the quality control of the series of measurements, Shewards cards were used for in-laboratory reference material (real sample). The analysis-to-analysis recovery was at the acceptable level of 80–120 %.

### Soil analysis

Twenty samples of forest soil collected from the Ap genetic horizon (depth of 0–20 cm) within the mushroom collection site with a soil auger were placed in air tight polypropylene containers. Greenery (grass or moss) and material other than soil (leaves, stones) were removed. Soil samples were transported to the laboratory directly after their collection. The soil samples were dried on Petri dishes for 48 h to determine water content according to Standard PN-ISO 11465:1999. Dry samples were transferred to an agate mortar and ground using a Cutting Boll Mill 200 (Retsch GmbH, Haan, Germany). Three subsamples (0.5 g) for each of 35 samples were extracted with aqua regia, according to standard PN-ISO 11466:2002. The soil was characterized by pH (PN-ISO 10390:1997), electrolytic conduction (PN-ISO 1265+AC1:1997), redox potential (ISO 11271:2002), and total nitrogen content using the Kjeldahl method. Content of K was analyzed by the Egner-Riehm method, Mg according to Schachtschabel’s method (Breś et al. 2009), while total organic carbon according to PN-ISO 14235:2003. Characteristics of soil where mushrooms were collected are shown in Table 2.

**Table 2:** Element concentration [mg kg^−1^ dry matter] and physico-chemical characteristics of soil in the sampling site and usual levels within Europe

Element	Concentration	Mean concentration in European soils*
Ag	0.29 ± 0.14	0.30 ± 0.23
Al**	2368 ± 329	10.50 ± 4.46***
As	0.74 ± 0.51	11.6 ± 20.1
Au	0.98 ± 0.35	nd
B	2.09 ± 0.88	nd
Ba	22.6 ± 3.49	400 ± 213
Bi	0.38 ± 0.17	nd
C	1.27 ± 0.31	2.48 ± 3.18
Ca**	0.03 ± 0.01	3.54 ± 7.26***
Cd	0.47 ± 0.31	0.28 ± 0.71
Co	1.64 ± 0.54	10.4 ± 13.3
Cr	4.12 ± 1.09	94.8 ± 285
Cu	3.26 ± 1.22	17.3 ± 19.0
Fe**	3263 ± 452	3.80 ± 2.34***
Ga	0.03 ± 0.02	13.1 ± 6.07
Ge	0.05 ± 0.03	nd
In	1.41 ± 0.76	0.05 ± 0.03
K**	0.04 ± 0.01	2.02 ± 0.95***
Li	0.76 ± 0.29	nd
Mg**	0.05 ± 0.02	1.18 ± 1.73***
Mn	112 ± 34	0.08 ± 0.07***
N	0.04 ± 0.03	nd
Na	186 ± 42	1.15 ± 0.95***
Ni	2.82 ± 1.05	37.3 ± 136
P**	0.03 ± 0.01	0.15 ± 0.12***
Pb	7.97 ± 2.87	32.6 ± 56.9
Re	0.21 ± 0.13	nd
Sb	0.19 ± 0.10	1.0 ± 2.0
Se	0.13 ± 0.07	nd
Sr	8.88 ± 3.14	130 ± 153
Te	0.62 ± 0.26	0.04 ± 0.05
Tl	0.13 ± 0.08	0.82 ± 1.02
Zn	15.39 ± 3.68	68 ± 141
Parameter	Unit	value
pH (H_2_O)	–	5.41 ± 0.09
Eh	mV	274 ± 42
Conductivity	mS m^−1^	89 ± 31

*nd* no data

^a^According to Geochemical Atlas of Europe (Salminen et al. 2005)

^b^Values in percent

^c^Calculated for Al_2_O_3_, CaO, Fe_2_O_3_, K_2_O, MgO, MnO, and P_2_O_5_, respectively

### Statistical analysis

In the present study, the effect of the experimental factor (mushroom species) on the accumulation of elements was assessed. The mean values of the analyzed variables (element contents) were compared for aboveground and wood-growing mushroom species separately. The Hotelling-Lawley test for MANOVA was used in the analysis of the simultaneous influence of many variables. One-way ANOVA with the Fisher’s test (*α* = 0.05) was used for evaluation of the effects of individual elements. Pairwise differences of mean element contents in the studied mushrooms were evaluated using Tukey’s method, which allowed the determination of homogenous groups (*α* = 0.01). Additionally, the Welch Two-Sample *t* test (*α* = 0.05) was applied to determine significance of differences between the average accumulation of individual elements.

Principal component analysis, PCA, was used in order to discover relationships between independent variables (element accumulation).

In addition, Heatmap Analysis with dendrograms based on hierarchical cluster analysis (HCA) was used to demonstrate similarities and differences in the accumulation of 26 elements in both the studied mushroom groups. To show the similarities or differences with regard to all 26 element contents jointly between mushroom species within both groups (aboveground and wood-growing mushrooms), the obtained results were illustrated using a Heatmap Analysis, where two dimension variables were represented by colors (Figs. 3 and 5). In a similar way, correlations between particular element contents were analyzed in aboveground and wood-growing mushroom groups (Figs. 4 and 6). Cluster Analysis allowed the selection of mushroom species (Figs. 3 and 5) and analyzed elements (Figs. 4 and 6) in the way that the relation between observations inside the same group was shown to be possibly the highest, while between different groups it was the lowest. Using Ward Hierarchical Clustering and Euclidean distances, tree diagrams with cluster grouping were obtained.

## Results and discussion

Overall data on the contents of 26 trace elements in 20 species are given in Table 3.

**Table 3:** Comparison of element contents [mg kg^−1^ d.w.] between mushrooms species inside of both groups (ANOVA) and between both groups (wood-growing species and aboveground mushroom species) using of Welch *t* test

Mushroom on wood	Ag	Al	As	Au	B	Ba	Bi	Cd	Co
*F* value	54.332	27.635	27.722	26.945	54.311	16.534	11.600	206.395	14.432
*A. mellea*	0.42b ± 0.08	24a ± 5	0.33ab ± 0.06	0.18bc ± 0.04	1.6bc ± 0.3	0.8cd ± 0.1	0.02b ± 0.01	0.69b ± 0.13	0.06bc ± 0.01
*A. auricula-judae*	0.18c ± 0.03	13bc ± 2	0.18bcd ± 0.03	0.03e ± 0.01	0.6bc ± 0.1	1.3bcd ± 0.2	0.03b ± 0.01	0.03b ± 0.01	0.02c ± 0.01
*F. velutipes*	0.21c ± 0.03	7cd ± 1	0.39a ± 0.04	0.06cd ± 0.01	0.1c ± 0.1	0.6d ± 0.1	0.03b ± 0.01	0.63b ± 0.08	0.15a ± 0.02
*G. applanatum*	0.06c ± 0.01	7cd ± 1	0.09d ± 0.02	0.33a ± 0.06	6.2a ± 1.2	2.9a ± 0.5	0.03b ± 0.01	0.12b ± 0.03	0.09b ± 0.02
*G. frondosa*	0.69a ± 0.08	25a ± 3	0.18cd ± 0.02	0.02e ± 0.01	2.1b ± 0.2	1.0cd ± 0.1	0.03b ± 0.01	8.01a ± 0.92	0.03c ± 0.01
*L. sulphureus*	0.21c ± 0.02	9bcd ± 1	0.33abc ± 0.04	0.03e ± 0.01	0.7bc ± 0.1	0.9cd ± 0.1	0.02b ± 0.01	0.06b ± 0.01	0.03c ± 0.01
*P. betulinus*	0.06c ± 0.02	2d ± 1	0.15d ± 0.05	0.03e ± 0.01	0.1c ± 0.1	1.9abc ± 0.6	0.02b ± 0.01	0.06b ± 0.02	0.06bc ± 0.02
*P. ostreatus*	0.09c ± 0.01	3cd ± 1	0.05d ± 0.01	0.12bcd ± 0.02	0.1c ± 0.1	0.4d ± 0.1	0.02b ± 0.01	0.12b ± 0.02	0.03c ± 0.01
*P.* sp.	0.21c ± 0.06	17ab ± 5	0.12d ± 0.03	0.33a ± 0.10	2.0b ± 0.6	2.6ab ± 0.8	0.02b ± 0.01	0.33b ± 0.10	0.09b ± 0.03
*P. squamosus*	0.15c ± 0.03	18ab ± 3	0.42a ± 0.08	0.21ab ± 0.03	5.9a ± 1.0	0.7cd ± 0.1	0.06a ± 0.01	0.09b ± 0.02	0.06bc ± 0.01
Mean	0.23	12.53	0.22	0.13	1.92	1.28	0.03	1.01	0.06
Mushroom at soil	Ag	Al	As	Au	B	Ba	Bi	Cd	Co
*F* value	32.913	43.715	21.980	47.840	42.676	74.912	3.224	23.751	26.988
*C. cibarius*	0.48b ± 0.18	49bc ± 18	0.57bc ± 0.21	0.01c ± 0.01	6.2b ± 2.3	1.1b ± 0.4	0.02a ± 0.01	0.21b ± 0.08	0.30a ± 0.11
*L. amethystina*	0.09b ± 0.01	11d ± 1	0.96bc ± 0.07	0.02c ± 0.01	1.2b ± 0.1	0.6b ± 0.1	0.03a ± 0.01	0.45b ± 0.03	0.03a ± 0.01
*L. scabrum*	0.03b ± 0.01	8d ± 1	0.57bc ± 0.10	0.02c ± 0.01	0.4b ± 0.1	0.4b ± 0.1	0.03a ± 0.01	0.27b ± 0.05	0.09a ± 0.02
*L. gilva*	1.83a ± 0.24	13d ± 2	0.36c ± 0.05	0.02c ± 0.01	4.8b ± 0.6	0.7b ± 0.1	0.02a ± 0.01	0.15b ± 0.02	0.03a ± 0.01
*L. fumosum*	0.24b ± 0.07	11d ± 3	0.48bc ± 0.15	0.09b ± 0.03	1.5b ± 0.5	0.7b ± 0.2	0.02a ± 0.01	1.05b ± 0.33	0.03a ± 0.01
*M. procera*	1.65a ± 0.53	11d ± 4	4.14a ± 1.33	0.02c ± 0.01	4.6b ± 1.5	0.5b ± 0.2	0.02a ± 0.01	4.71a ± 1.51	0.15a ± 0.05
*P. involutus*	1.92a ± 0.40	7d ± 1	0.54bc ± 0.11	0.03c ± 0.01	22.6a ± 4.7	0.5b ± 0.1	0.03a ± 0.01	0.12b ± 0.03	0.03a ± 0.01
*S. bovinus*	0.18b ± 0.02	22cd ± 3	1.95b ± 0.22	0.15a ± 0.02	4.3b ± 0.5	0.5b ± 0.1	0.03a ± 0.01	1.08b ± 0.12	0.02a ± 0.01
*S. luteus*	0.08b ± 0.01	107a ± 11	0.57bc ± 0.06	0.03c ± 0.01	1.5b ± 0.2	3.9a ± 0.4	0.03a ± 0.01	0.09b ± 0.01	0.36a ± 0.04
*T. equestre*	0.48b ± 0.12	70b ± 18	0.33c ± 0.08	0.02c ± 0.01	0.3b ± 0.1	0.8b ± 0.2	0.02a ± 0.01	0.81b ± 0.21	0.09a ± 0.02
Mean	0.70	30.94	1.05	0.04	4.74	0.95	0.03	0.89	0.11
Welch *t* test	−1.86	−1.67	−2.18*	2.28*	−1.27	0.77	0.93	0.13	−1.27
Mushroom on wood	Cr	Cu	Fe	Ga	Ge	In	Li	Mn	Ni
*F* value	10.573	72.235	30.509	7.667	4.611	26.422	26.814	26.371	91.760
*A. mellea*	0.12abc ± 0.03	8.0cd ± 1.6	57b ± 11	0.02ab ± 0.01	0.02a ± 0.01	0.08bc ± 0.02	0.05b ± 0.01	7.7b ± 1.5	0.06cd ± 0.01
*A. auricula-judae*	0.09abc ± 0.02	2.5fg ± 0.4	37bc ± 6	0.02ab ± 0.01	0.03a ± 0.01	0.05bc ± 0.01	0.03b ± 0.01	2.2b ± 0.3	0.12bcd ± 0.02
*F. velutipes*	0.09bcd ± 0.01	22.7a ± 2.8	21cd ± 3	0.01b ± 0.01	0.03a ± 0.01	0.09bc ± 0.01	0.02b ± 0.01	5.2b ± 0.6	0.66a ± 0.08
*G. applanatum*	0.06cd ± 0.01	9.4bc ± 1.7	22cd ± 4	0.02ab ± 0.01	0.02a ± 0.01	0.21a ± 0.04	0.06b ± 0.01	15.5b ± 2.9	0.12bcd ± 0.03
*G. frondosa*	0.09bcd ± 0.01	13.1b ± 1.5	87a ± 10	0.03a ± 0.01	0.03a ± 0.01	0.07bc ± 0.01	0.03b ± 0.01	12.6b ± 1.4	0.18b ± 0.02
*L. sulphureus*	0.09abc ± 0.01	2.1fg ± 0.2	28cd ± 3	0.02ab ± 0.01	0.03a ± 0.01	0.09bc ± 0.01	0.02b ± 0.01	2.4b ± 0.3	0.03d ± 0.01
*P. betulinus*	0.03d ± 0.01	1.2g ± 0.4	5d ± 2	0.02ab ± 0.01	0.03a ± 0.01	0.12b ± 0.04	0.01b ± 0.01	2.5b ± 0.9	0.03d ± 0.01
*P. ostreatus*	0.15a ± 0.02	2.4fg ± 0.3	43bc ± 5	0.02ab ± 0.01	0.02a ± 0.01	0.06bc ± 0.01	0.07b ± 0.01	3.5b ± 0.4	0.12bcd ± 0.02
*P.* sp.	0.09abc ± 0.03	3.8def ± 1.1	39bc ± 11	0.02ab ± 0.01	0.02a ± 0.01	0.03c ± 0.01	0.29a ± 0.07	39.0a ± 11.5	0.15bc ± 0.05
*P. squamosus*	0.12ab ± 0.02	6.3cde ± 1.2	34bc ± 6	0.01b ± 0.01	0.03a ± 0.01	0.37a ± 0.05	0.02b ± 0.01	3.9b ± 0.8	0.15bc ± 0.03
Mean	0.09	7.15	37.41	0.02	0.03	0.11	0.06	9.45	0.16
Mushroom at soil	Cr	Cu	Fe	Ga	Ge	In	Li	Mn	Ni
*F* value	222.934	23.601	155.263	48.409	4.332	17.673	185.019	11.736	24.547
*C. cibarius*	0.12b ± 0.04	18.7bc ± 6.9	37c ± 14	0.02b ± 0.01	0.03a ± 0.01	0.07c ± 0.03	0.02b ± 0.01	27.6a ± 10.1	1.20a ± 0.44
*L. amethystina*	0.09b ± 0.01	4.0c ± 0.3	41c ± 3	0.01b ± 0.01	0.03a ± 0.01	0.19ab ± 0.02	0.03b ± 0.01	9.3c ± 0.7	0.24b ± 0.02
*L. scabrum*	0.09b ± 0.02	19.8bc ± 3.4	20c ± 3	0.01b ± 0.01	0.02a ± 0.01	0.18ab ± 0.03	0.02b ± 0.01	5.7c ± 1.0	0.18b ± 0.03
*L. gilva*	0.12b ± 0.02	12.4c ± 1.6	24c ± 3	0.02b ± 0.01	0.03a ± 0.01	0.18ab ± 0.02	0.03b ± 0.01	11.9bc ± 1.6	0.15b ± 0.02
*L. fumosum*	0.12b ± 0.04	9.3c ± 2.9	36c ± 1	0.02b ± 0.01	0.02a ± 0.01	0.09c ± 0.03	0.09b ± 0.03	8.2c ± 2.5	0.03b ± 0.01
*M. procera*	0.12b ± 0.04	132.5a ± 42.4	32c ± 10	0.03b ± 0.01	0.03a ± 0.01	0.12bc ± 0.04	0.02b ± 0.01	26.3ab ± 8.4	0.09b ± 0.03
*P. involutus*	0.15b ± 0.03	66.2b ± 13.9	28c ± 6	0.02b ± 0.01	0.03a ± 0.01	0.12bc ± 0.03	0.02b ± 0.01	2.7c ± 0.6	0.09b ± 0.02
*S. bovinus*	0.09b ± 0.01	7.7c ± 0.9	621a ± 72	0.09a ± 0.01	0.03a ± 0.01	0.12bc ± 0.01	0.02b ± 0.01	4.7c ± 0.5	0.36b ± 0.04
*S. luteus*	2.01a ± 0.20	5.6c ± 0.6	315b ± 31	0.01b ± 0.01	0.02a ± 0.01	0.26a ± 0.02	0.56a ± 0.06	15.4abc ± 1.5	1.02a ± 0.10
*T. equestre*	0.15b ± 0.04	15.9bc ± 4.1	111c ± 28	0.02b ± 0.01	0.01a ± 0.01	0.06c ± 0.02	0.09b ± 0.02	8.5c ± 2.2	0.12b ± 0.03
Mean	0.31	29.21	126.32	0.03	0.03	0.14	0.09	12.02	0.35
Welch *t* test	−1.12	−1.70	−1.43	−0.73	0.70	−1.04	−0.60	−0.57	−1.30
Mushroom on wood	Pb	Re	Sb	Se	Sr	Te	Tl	Zn	
*F* value	13.680	5.589	23.547	38.510	32.358	10.710	21.189	12.663	
*A. mellea*	0.21cde ± 0.04	0.09a ± 0.02	0.03c ± 0.01	0.36bc ± 0.07	0.6c ± 0.1	0.15abc ± 0.03	0.09a ± 0.02	43bcd ± 8	
*A. auricula-judae*	0.45abcd ± 0.07	0.09a ± 0.02	0.03c ± 0.01	0.27bc ± 0.04	2.7b ± 0.4	0.09bc ± 0.02	0.02b ± 0.01	16d ± 3	
*F. velutipes*	0.36bcde ± 0.04	0.06a ± 0.01	0.03c ± 0.01	0.42ab ± 0.05	1.0bc ± 0.1	0.27a ± 0.03	0.02b ± 0.01	43bcd ± 5	
*G. applanatum*	0.27bcde ± 0.05	0.06a ± 0.01	0.15a ± 0.03	0.06d ± 0.01	5.2a ± 1.0	0.09bc ± 0.02	0.01b ± 0.01	45bcd ± 9	
*G. frondosa*	0.24cde ± 0.03	0.06a ± 0.01	0.03c ± 0.01	0.39b ± 0.05	0.5c ± 0.1	0.21ab ± 0.03	0.02b ± 0.01	28cd ± 3	
*L. sulphureus*	0.48abc ± 0.06	0.09a ± 0.01	0.06bc ± 0.01	0.09d ± 0.01	1.3bc ± 0.2	0.15abc ± 0.02	0.02b ± 0.01	29cd ± 3	
*P. betulinus*	0.18de ± 0.06	0.12a ± 0.04	0.06bc ± 0.02	0.09d ± 0.03	0.5c ± 0.2	0.21abc ± 0.07	0.02b ± 0.01	78ab ± 26	
*P. ostreatus*	0.15e ± 0.02	0.06a ± 0.01	0.09b ± 0.01	0.21cd ± 0.03	0.4c ± 0.1	0.18abc ± 0.03	0.02b ± 0.01	90a ± 11	
*P.* sp.	0.69a ± 0.20	0.06a ± 0.02	0.06bc ± 0.02	0.06d ± 0.02	4.7a ± 1.4	0.09c ± 0.03	0.09a ± 0.03	66abc ± 20	
*P. squamosus*	0.54ab ± 0.10	0.12a ± 0.02	0.06bc ± 0.01	0.57a ± 0.10	0.5c ± 0.1	0.24a ± 0.04	0.02b ± 0.01	28cd ± 5	
Mean	0.36	0.08	0.06	0.25	1.73	0.17	0.03	46.57	
Mushroom at soil	Pb	Re	Sb	Se	Sr	Te	Tl	Zn	
*F* value	21.735	14.528	24.000	10.467	127.235	6.144	34.568	12.668	
*C. cibarius*	0.30b ± 0.11	0.06bc ± 0.02	0.02b ± 0.01	0.90abc ± 0.33	0.6b ± 0.2	0.27ab ± 0.10	0.02b ± 0.01	107abc ± 39	
*L. amethystina*	0.36b ± 0.03	0.06bc ± 0.01	0.03b ± 0.01	0.78bc ± 0.05	0.4b ± 0.1	0.24ab ± 0.02	0.02b ± 0.01	35c ± 2	
*L. scabrum*	0.27b ± 0.05	0.02c ± 0.01	0.09a ± 0.02	1.38ab ± 0.24	0.2b ± 0.1	0.27ab ± 0.05	0.02b ± 0.01	92bc ± 16	
*L. gilva*	0.42b ± 0.06	0.03c ± 0.01	0.02b ± 0.01	1.50ab ± 0.20	0.6b ± 0.1	0.09b ± 0.01	0.02b ± 0.01	53bc ± 7	
*L. fumosum*	0.12 b ± 0.04	0.09bc ± 0.03	0.03b ± 0.01	0.45c ± 0.14	0.9b ± 0.3	0.18ab ± 0.06	0.03b ± 0.01	42c ± 13	
*M. procera*	2.52a ± 0.81	0.12b ± 0.04	0.03b ± 0.01	1.62a ± 0.52	0.5b ± 0.2	0.15ab ± 0.05	0.02b ± 0.01	71bc ± 23	
*P. involutus*	0.18b ± 0.04	0.09bc ± 0.02	0.03b ± 0.01	0.75bc ± 0.16	0.4b ± 0.1	0.27ab ± 0.06	0.02b ± 0.01	136ab ± 28	
*S. bovinus*	0.12b ± 0.01	0.12b ± 0.01	0.09a ± 0.01	0.72bc ± 0.08	0.4b ± 0.1	0.30a ± 0.03	0.02b ± 0.01	53bc ± 6	
*S. luteus*	0.54b ± 0.05	0.06bc ± 0.01	0.03b ± 0.01	0.54c ± 0.05	4.4a ± 0.4	0.12ab ± 0.01	0.02b ± 0.01	43c ± 4	
*T. equestre*	0.66b ± 0.17	0.21a ± 0.05	0.09a ± 0.02	0.45c ± 0.11	0.5b ± 0.1	0.27ab ± 0.07	0.21a ± 0.05	186a ± 47	
Mean	0.55	0.09	0.05	0.91	0.88	0.22	0.04	81.73	
Welch *t* test	−0.83	−0.19	0.92	−4.41**	1.22	−1.55	−0.35	−2.04	

Mean values (*n* = 3) ± standard deviations; identical letters (a, b, c….) denote no significant (*p* < 0.05) difference between mean values in column according to Tukey’s HDS test (ANOVA)

*p < 0.05; **p < 0.01 (Welch Two-Sample *t* test)

Trace elements in mushrooms have been considered mainly from two perspectives. The environmental view concerns factors that affect bioaccumulation of individual trace elements in both mycelium and fruit bodies, particularly the role of such processes within mycorrhiza. Attempts to utilize the fruit bodies of some species as biomarkers of local contamination with various trace elements culminated during the 1980s and was gradually abandoned with no selection of any credible species (Wondratschek and Röder 1993). The second perspective deals with health and nutritional effects, in particular the detrimental elements Cd, Hg, Pb, Ag, and As in edible species. A connected aspect, the content of nutritionally required trace elements (particularly Se), has been problematic due to a virtual lack of knowledge of their bioaccessibility and bioavailability in humans.

There occur significant inter-year differences in trace metal contents within individual mushroom species. This has repeatedly been observed in mushrooms collected from the same site over the course of several years, e.g., by Chojnacka et al. (2013) in *Xerocomus subtomentosus*, Chudzyński et al. (2013) in *Suillus grevillei*, Gucia et al. (2012b) in *Macrolepiota procera*, and Zhang et al. (2010) in *Boletus edulis*. The cited works are based on an assumption that fruit bodies are formed by a steady mycelium. However, more mycelia of the same species can be present at the sampling site. A hypothesis was put forward that element levels in wild growing mushrooms considerably elevate with the increasing age of mycelium and protracted time lag between the fructifications (Kalač 2010).

Comparing the mean contents of both the mushroom groups gathered in Table 3, it can be seen that the aboveground species had a significantly higher mean level of 12 elements (Ag, Al, As, B, Co, Cr, Cu, Fe, Ni, Pb, Se, and Zn) than the wood-growing mushrooms. An opposite relation was observed for Au, Ba, and Sr. Insignificant differences were found in the 11 remaining elements (Bi, Cd, Ga, Ge, In, Li, Mn, Re, Sb, Te, and Tl). Nevertheless, it should be borne in mind that some mean contents are distorted by outlying values, e.g., of As in *M. procera* (4.14 ± 1.33 mg kg^−1^ d.m.) or Fe in *Suillus luteus* (315 ± 31.4 mg kg^−1^ d.m.). It is not possible to compare the results with the literature data because of a lack of information on the comparison of both the groups.

Based on a variance analysis at *α* = 0.05, separately for wood-growing species and aboveground mushroom species, it was stated that similarities and differences between them were diverse. The detailed Tukey test made it possible to select significantly different mushroom species as regards their abilities to accumulate particular elements inside the particular groups (Table 3).

No significant differences between the ability of mushroom species to accumulate germanium were observed in either of the groups. For most of the tested elements (Ag, Al, As, Au, B, Ba, Cd, Cr, Cu, Fe, Ga, In, Li, Mn, Ni, Pb, Sb, Se, Sr, Te, Tl, and Zn), significant differences (*α* = 0.01) in the accumulation capacities in both the groups (wood-growing and aboveground mushroom species) were observed. Accumulation of Bi and Co was only diverse within the wood-growing species, while Re accumulation in the aboveground mushroom species only. When we compare both the groups of mushrooms, significant differences in As, Au (*α* = 0.05), and Se (*α* = 0.01) accumulation were revealed between the groups on the basis of the Welch Two-Sample *t* test.

Literature data on trace elements for most of the tested wood-growing species are very limited, dealing preferably with *Armillaria mellea*, *Flammulina velutipes*, *Pleurotus ostreatus* and sporadically with *Laetiporus sulphureus* (Durkan et al. 2011; Gramss and Voigt 2013; Huang et al. 2015; Kaya and Bag 2010; Sarikurkcu et al. 2012; Severoglu et al. 2013; Zeng et al. 2012; Zhu et al. 2011). Moreover, the reported trace elements are considerably limited when compared to the present work. Overall, the literature data are comparable with our results only for Zn, while for numerous elements (Al, As, B, Co, Cr, Cu, Fe, Mn, Ni, and Pb) the reported levels are considerably higher, often by 1 order of magnitude. Cadmium contents fluctuated. Literature information on bioconcentration factors (BCF), the ratio of metal content in a mushroom fruit body to the content in wood substrate (both in mg kg^−1^ d.m.), is virtually lacking.

The mean contents of most individual elements in the group of aboveground species can be compared with extensive available data, particularly those collated in the review of Kalač (2010). In general, similar contents of As, B, Cd, Cu, Fe, Ga, Mn, Ni, Sb, Sr, and Zn were determined in comparison with the contents reported in fruit bodies of numerous mushroom species collected from unpolluted sites. The levels of Ag, Al, Ba, Co, Cr, Pb, Se, and Tl were lower than usually reported in the literature data, while the opposite relation was observed only for Au and Li. Literature data for Bi, Ge, In, Re, and Te are still insufficient.

Cuny et al. (2001) reported increased contents of cadmium, lead, and zinc in fruit bodies of several mushroom species growing along a French motorway as compared with the usual level for the same species collected from unpolluted sites. No similar situation was observed within the data of Table 3. This also deals with Pb, which has formerly contaminated areas adjacent to heavily frequented roads from leaded petrol. Leaded petrol has not been used in Poland since the end of 2004.

**Fig. 2:**
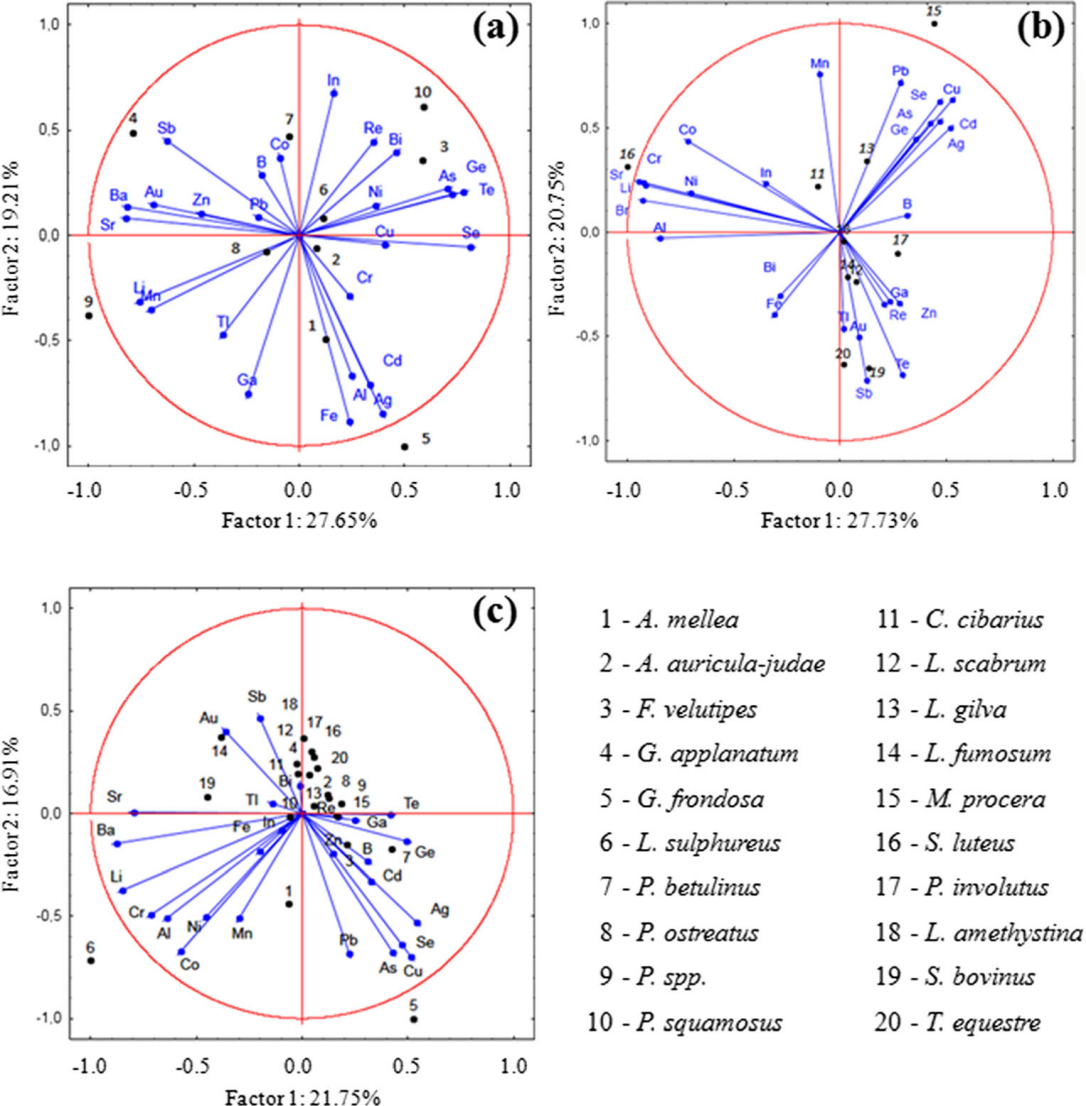
PCA analysis for tested wood-growing species (**a**), aboveground mushroom species (**b**), and for both groups of mushrooms (**c**)

For a graphical presentation of the obtained results and relationships between tested mushroom species growing on wood and in soil, a PCA was performed (Fig. 2). The analyses were performed for aboveground and wood-growing mushroom species separately and the obtained results allowed similarities and differences in element accumulation to be determined in the studied mushroom species.

In mushrooms growing on wood (Fig. 2a), PCA analysis explained 46.86 % of total variability of mushrooms to the observed element accumulation. Obtained results suggested that accumulation of Ag, Al, Cd, and Fe was independent of As, Ge, Se, and Te as well as Li and Mn. Simultaneously, some groups of elements predominantly influenced element accumulation in individual mushroom species. In wood-growing species, the following relationships were observed: *Polyporus squamosus* and *Flammulina velutipes* in relation to Bi, In, and Re and also As, Ge, Te, and Se; *Ganoderma applanatum*—Au, Ba, and Sr as well as Sb; *Pleurotus* spp.—Li and Mn; *Grifola frondosa*—Ag, Al, Cd, and Fe (Fig. 2a).

In mushrooms growing in soil (Fig. 2b), two principal components explained 48.48 % of total variability. Accumulation of Au, Ga, Re, Sb, Te, Tl, and Zn was independent of Ag, As, Cd, Cu, Ge, Pb, and Se. Additionally, accumulation of the last group of elements was independent of Al, Ba, Co, Cr, Li, Ni, and Sr. In the aboveground species, the following relationships were stated: *Macrolepiota procera—*As, Ag, Cd, Cu, Ge, Pb, and Se; *Suillus luteus*—Al, Ba, Co, Cr, Li, Ni, and Sr; *Suillus bovinus* and *Tricholoma equestre*—Au, Ga, Re, Te, Tl, Sb, and Zn (Fig. 2b).

In the file of all the analyzed mushrooms and elements (Fig. 2c), two principal components explained 38.66 % of total variability. Accumulation of Ag, As, Cu, Pb, and Se was independent of Al, Co, Cr, Li, Ni, and Mn. Based on all 20 tested mushroom species and all 26 analyzed elements, the following relationships within both the groups were as follows: *Grifola frondosa*—Ag, As, Cu, Pb, and Se; *Laetiporus sulphureus*—Al, Co, Cr, Li, Ni, and Mn.

### Content of elements in wood-growing species

Varying contents of elements were observed within the group of wood-growing species. As may be seen in the results from Table 3, each of the four species displayed the highest level of five elements: *Ganoderma applanatum* (Au, B, Ba, Sb, and Sr), *Grifola frondosa* (Ag, Al, Cd, Fe, and Ga), *Pleurotus* spp. (Au, Li, Mn, Pb, and Tl), and *Polyporus squamosus* (As, Bi, In, Re, and Se). *Armillaria mellea* displayed a high content of Al (24 ± 5 mg kg^−1^ d.m.); its mean Tl level was comparable with *Pleurotus* spp. (0.09 mg kg^−1^ d.m.). *F. velutipes* contained elevated levels of As, Co, Cu, and Ni (0.39 ± 0.04, 0.15 ± 0.02, 22.7 ± 2.8, and 0.66 ± 0.08 mg kg^−1^ d.m., respectively). The highest content of Cr and Zn was observed in fruit bodies of *Pleurotus ostreatus* (0.15 ± 0.02 and 90 ± 11.2 mg kg^−1^ d.m., respectively). The greatest of all the significant differences among the tested species were observed for Pb, Cr, and Cu, while no differences were found for Ge and Re. Information on trace elements in wood-growing species is very fragmentary in the literature.

**Fig. 3:**
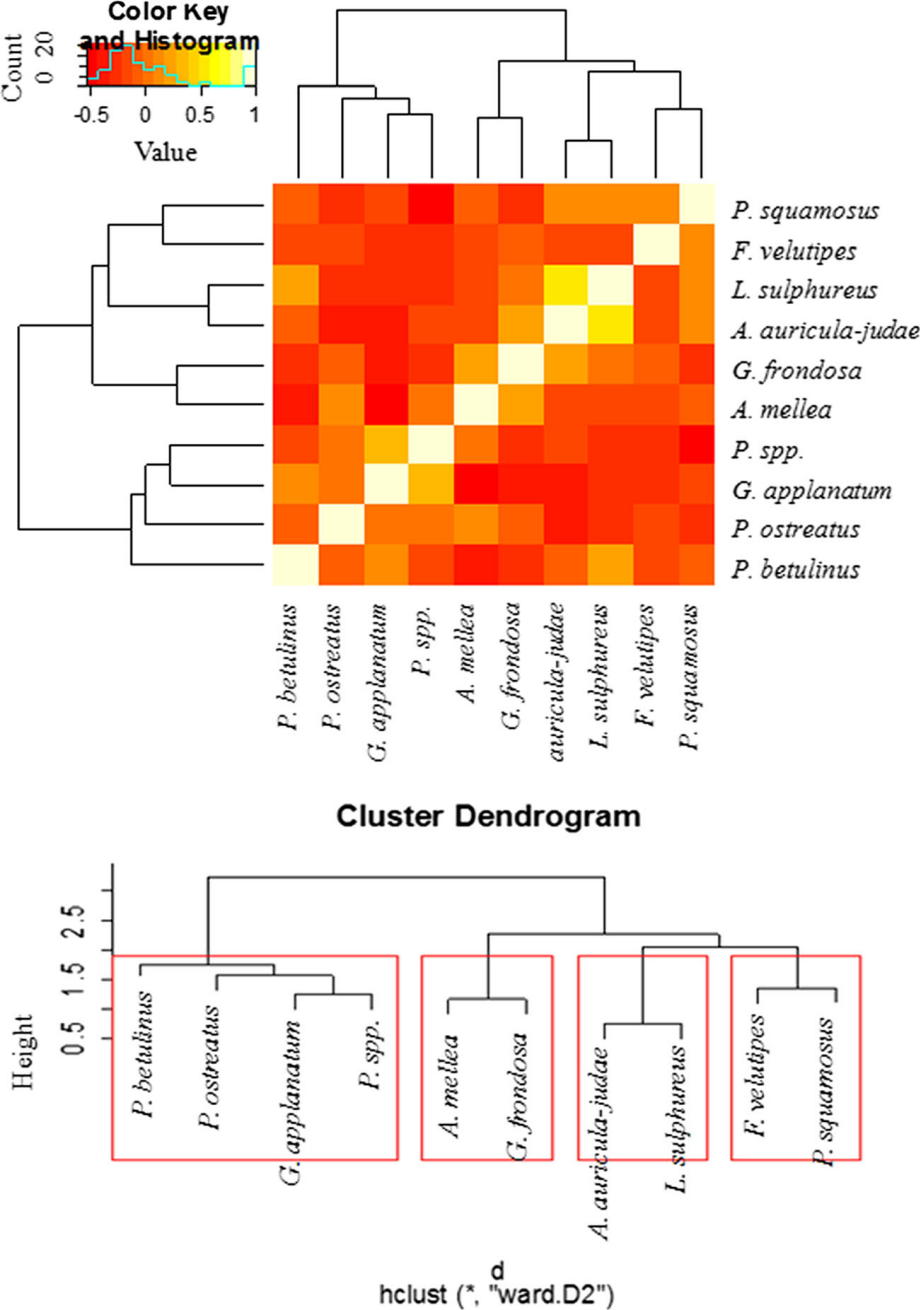
Correlation between tested wood-growing species with regard to the accumulation of all analyzed elements jointly (Heatmap) with presentation of a hierarchical tree plot to show the groups of mushrooms characterized by a high similarity to all element accumulation

Based on the obtained results, the correlation between the tested mushroom species growing on wood as regards the accumulation of all analyzed elements jointly (Heatmap) reveals similarities and differences between mushroom species (Fig. 3).

**Fig. 4:**
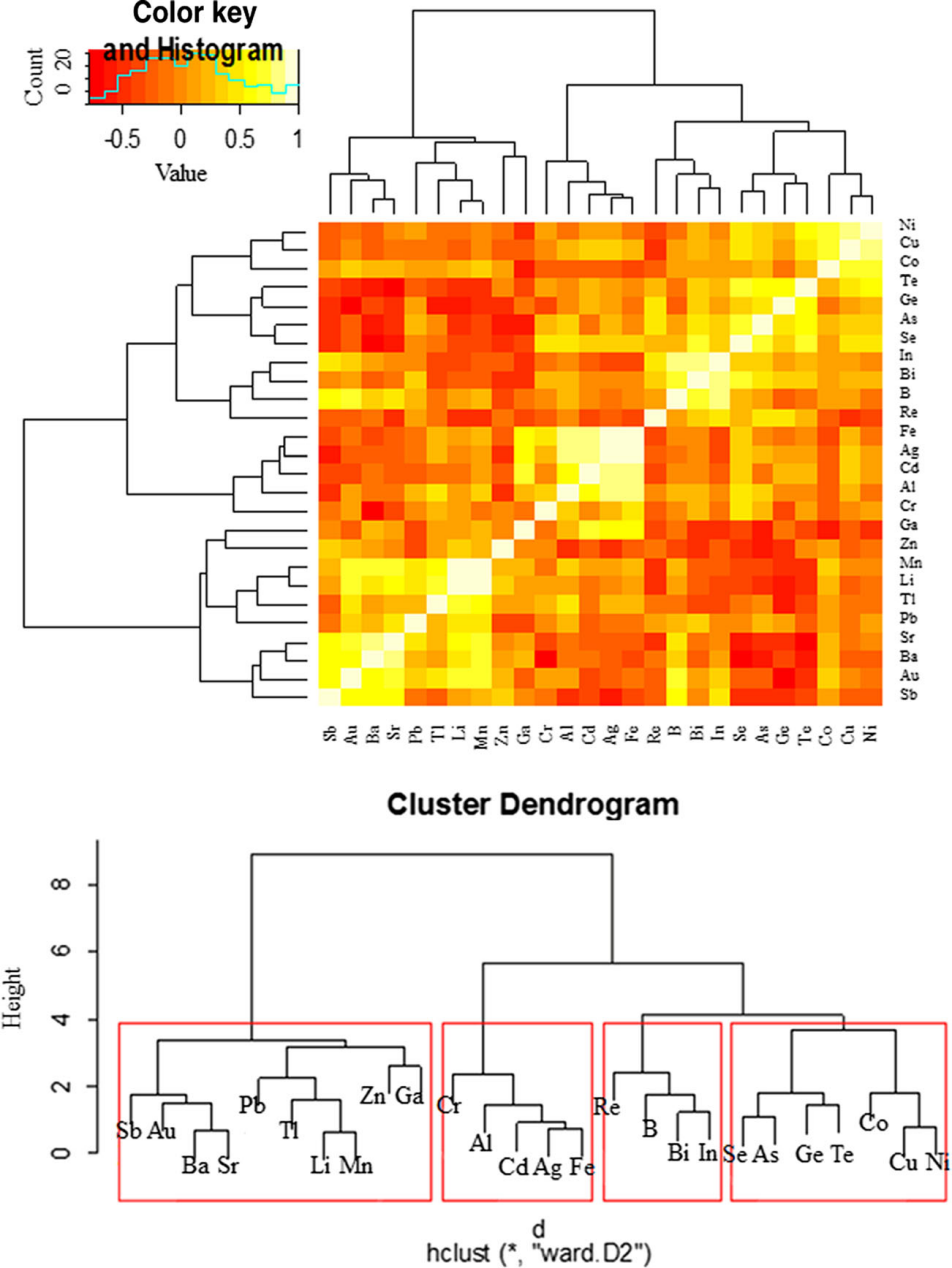
Correlation between analyzed elements as regards their accumulation in all wood-growing species (Heatmap) with presentation of a hierarchical tree plot to show the groups of similarly accumulated elements

A cluster dendrogram was also performed so as to distinguish which mushroom groups accumulate elements in a similar way. Four diverse groups of mushrooms were indicated: (i) *Piptoporus betulinus*, *Pleurotus ostreatus*, *Ganoderma applanatum*, and *Pleurotus* spp., (ii) *Armillaria mellea* and *Grifola frondosa*; (iii) *Auricularia auricula-judae* and *Laetiporus sulphureus*; and (iv) *F. velutipes* and *Polyporus squamosus*. On the other hand, in order to show the similarities between elements, which were accumulated in the same way by all wood-growing species, another Heatmap analysis was performed (Fig. 4).

In this case, four groups of elements were indicated: (i) Au, Ba, Li, Ga, Mn, Pb, Sb, Sr, Tl, and Zn; (ii) Al, Ag, Cd, Cr, and Fe; (iii) B, Bi, In, and Re; (iv) As, Co, Cu, Ge, Ni, Se, and Te.

### Content of elements in aboveground mushroom species

The tested mushroom species were diverse in terms of their ability to accumulate particular elements from soil (Table 3). Some species were able to selectively accumulate particular elements and limit the accumulation of others. *Cantharellus cibarius* was found to accumulate the highest content of Mn and Ni (27.6 ± 10.1 and 1.20 ± 0.44 mg kg^−1^ d.m., respectively), while *M. procera* fruit bodies contained the highest levels of As, Cd, Cu, Pb, and Se (4.14 ± 1.33, 4.71 ± 1.51, 132.5 ± 42.4, 2.52 ± 0.81, and 1.62 ± 0.52 mg kg^−1^ d.m., respectively). *M. procera* is known to be an accumulator of these elements (Kalač 2010). The highest content of Ag was observed in *Lepista gilva*, *M. procera*, and *Paxillus involutus* (1.83 ± 0.24, 1.65 ± 0.53, and 1.92 ± 0.40 mg kg^−1^ d.m., respectively). *Paxillus involutus* was the most effective boron accumulator (22.6 ± 4.7 mg kg^−1^ d.m.) among mushroom species. For other elements, the highest content of Al, Ba, Cr, In, Li, and Sr was observed in *S. luteus* fruit bodies (107 ± 11, 3.9 ± 0.4, 2.01 ± 0.20, 0.26 ± 0.02, 0.56 ± 0.06, and 4.4 ± 0.4 mg kg^−1^ d.m., respectively), while As, Fe, Ga, and Te in *S. bovinus* (0.15 ± 0.02, 621 ± 72, 0.09 ± 0.01, and 0.30 ± 0.03 mg kg^−1^ d.m., respectively). The highest content of Re, Tl, and Zn was observed in *T. equestre* (0.21 ± 0.05, 0.21 ± 0.05, and 186 ± 47 mg kg^−1^ d.m., respectively). In three elements, Bi, Co, and Ge, no significant differences were observed among the tested mushroom species.

**Fig. 5:**
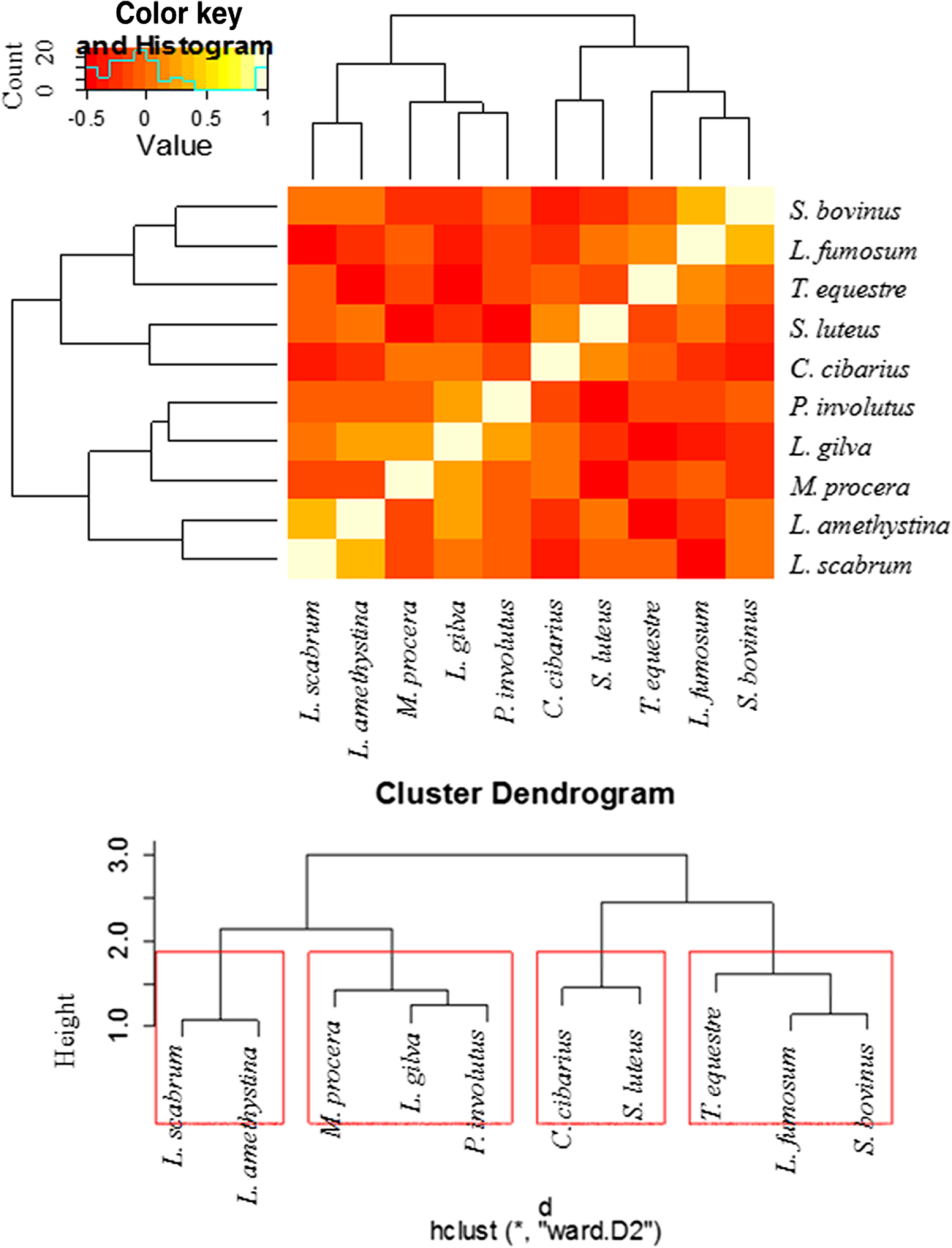
Correlation between tested aboveground mushroom species with regard to the accumulation of all analyzed elements jointly (Heatmap) with presentation of a hierarchical tree plot to show the groups of mushrooms characterized by a high similarity to all element accumulation

Analysis of the Heatmap in order to compare the tested aboveground mushroom species as regards their abilities to accumulate all 26 elements pointed to the existence of four different mushroom groups (Fig. 5). The following mushroom species were included in the specified groups: (i) *Leccinum scabrum* and *Laccaria amethystina*; (ii) *M. procera*, *Lepista gilva*, and *Paxillus involutus*; (iii) *C. cibarius* and *S. luteus*; and (iv) *T. equestre*, *Lyophyllum fumosum* and *S. bovinus*.

**Fig. 6:**
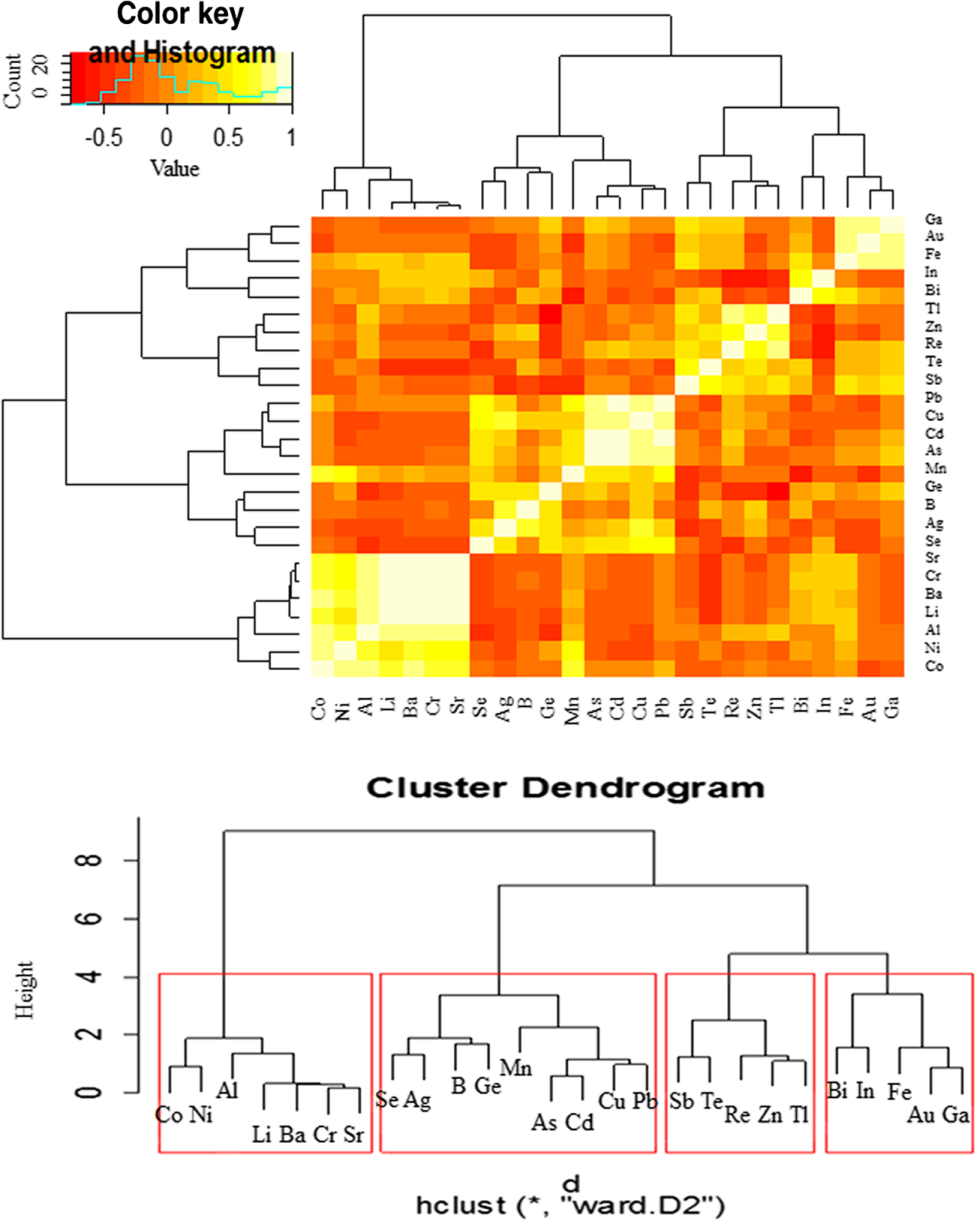
Correlation between analyzed elements as regards their accumulation in all aboveground mushroom species (Heatmap) with presentation of a hierarchical tree plot to show the groups of similarly accumulated elements

In the additional Heatmap analysis, the correlation between each of the analyzed elements considering their accumulation in all tested mushroom species growing in soil is presented below (Fig. 6). The data pointed to the existence of four variable groups of elements. The elements were accumulated within each of the groups by all mushroom species in the same or a similar way. Based on a hierarchical tree plot, the elements were classified into four groups: (i) Al, Ba, Co, Cr, Li, Ni, and Sr; (ii) Ag, As, B, Cd, Cu, Ge, Mn, Pb, and Se; (iii) Re, Sb, Te, Tl, and Zn; and (iv) Au, Bi, Fe, Ga, and In.

To show the efficiency of the tested mushroom species to element uptake, the BCF values were calculated (Table 4).

**Table 4:** Bioconcentration factor (BCF) values for aboveground mushroom species

Element	*C. cibarius*	*L. amethystina*	*L. scabrum*	*L. gilva*	*L. fumosum*	*M. procera*	*P. involutus*	*S. bovinus*	*S. luteus*	*T. equestre*
Ag	1.66	0.31	0.10	6.31	0.83	5.69	6.62	0.62	0.28	1.66
Al	0.02	0.00	0.00	0.01	0.00	0.00	0.00	0.01	0.05	0.03
As	0.77	1.30	0.77	0.49	0.65	5.59	0.73	2.64	0.77	0.45
Au	0.01	0.02	0.02	0.02	0.09	0.02	0.03	0.15	0.03	0.02
B	2.99	0.59	0.17	2.30	0.72	2.20	10.81	2.04	0.72	0.16
Ba	0.05	0.03	0.02	0.03	0.03	0.02	0.02	0.02	0.17	0.03
Bi	0.05	0.08	0.08	0.05	0.05	0.05	0.08	0.08	0.08	0.05
Cd	0.45	0.96	0.57	0.32	2.23	10.02	0.26	2.30	0.19	1.72
Co	0.18	0.02	0.05	0.02	0.02	0.09	0.02	0.01	0.22	0.05
Cr	0.03	0.02	0.02	0.03	0.03	0.03	0.04	0.02	0.49	0.04
Cu	5.74	1.21	6.08	3.81	2.85	40.63	20.31	2.36	1.72	4.88
Fe	0.01	0.01	0.01	0.01	0.01	0.01	0.01	0.19	0.10	0.03
Ga	0.67	0.33	0.33	0.67	0.67	1.00	0.67	3.00	0.33	0.67
Ge	0.60	0.60	0.40	0.60	0.40	0.60	0.60	0.60	0.40	0.20
In	0.05	0.17	0.13	0.13	0.06	0.09	0.09	0.09	0.15	0.04
Li	0.03	0.04	0.03	0.04	0.12	0.03	0.03	0.03	0.73	0.12
Mn	0.25	0.08	0.05	0.11	0.07	0.24	0.02	0.04	0.14	0.08
Ni	0.43	0.09	0.06	0.05	0.01	0.03	0.03	0.13	0.36	0.04
Pt	0.04	0.05	0.03	0.05	0.02	0.32	0.02	0.02	0.07	0.08
Re	0.29	0.29	0.10	0.14	0.43	0.57	0.43	0.57	0.29	1.00
Sb	0.11	0.16	0.47	0.11	0.16	0.16	0.16	0.47	0.16	0.47
Se	6.92	6.00	10.62	11.54	3.46	12.46	5.77	5.54	4.15	3.46
Sr	0.06	0.05	0.03	0.06	0.10	0.05	0.04	0.04	0.50	0.06
Te	0.44	0.39	0.44	0.15	0.29	0.24	0.44	0.48	0.19	0.44
Tl	0.15	0.15	0.15	0.15	0.23	0.15	0.15	0.15	0.15	1.62
Zn	6.97	2.26	5.97	3.42	2.74	4.64	8.81	3.47	2.76	12.06

BCF >1 affirmed that element accumulation took place in (was observed for) all 10 aboveground mushroom species in the case of Cu, Se, and Zn. Moreover, the same observations were stated for *C. cibarius* (Ag, B), *Laccaria amethystina* (As), *Lepista gilva* (Ag, B), *Lyophyllum fumosum* (Cd), *M. procera* (Ag, As, B, and Cd), *Paxillus involutus* (Ag, B), *S. bovinus* (As, B, Cd, and Ga), and *T. equestre* (Ag, Cd, Re, and Tl). For the remaining elements in analyzed mushroom species, BCF values were lower than 1, which was related with their bioexclusion. Alonso et al. (2003) have also analyzed *C. cibarius*, *Leccinum scabrum*, *M. procera*, and *T. equestre* and calculated BCF values for Cu (3.30, 2.94, 18.64, and 6.25, respectively) and Zn (5.63, 4.96, 3.45, and 6.17, respectively). Their values were generally similar to those presented in this paper (5.74, 6.08, 40.63, and 4.88, respectively for Cu and 6.97, 5.97, 4.64, and 12.06, respectively for Zn). Additionally, presented data confirmed observations reported by Gucia et al. (2012a), who pointed out the bioexclusion of Al, Ba, Co, Cr, Fe, Ni, and Sr and the effective accumulation of Ag, Cd, Cu, and Zn in *M. procera* fruiting bodies.

### Intake of mushrooms and the risk for humans

A recent recommendation of the WHO sets a provisional tolerable monthly intake (PTMI) for cadmium at 0.025 mg kg^−1^ bodyweight for an adult. For an individual weighing 60 kg, the value is 1.5 mg per month. An average single serving is 300 g of fresh mushrooms, i.e., about 30 g of d.m., as a 10 % level is used for calculations with an unknown factual d.m. level. A less probable scenario is a serving of 500 g. In *Grifola frondosa*, the species with the highest mean Cd content of 8.01 mg kg^−1^ d.m., the intake would be 0.24 and 0.40 mg from lower and higher model serving, respectively. The respective intakes from *M. procera*, with a mean content in the present work of 4.71 mg Cd per kg d.m., the respective intake would be 0.14 and 0.24 mg.

The WHO determines the maximum daily intake of lead at 0.0012 mg kg^−1^ bodyweight, i.e., 0.072 mg for an adult weighing 60 kg. The maximum determined Pb content of 2.52 mg kg^−1^ d.m. in *M. procera* would thus deliver a dose of 0.076 and 0.126 mg from servings of 300 and 500 g of the fresh *M. procera*, respectively.

Considering the above calculations, a potential health risk would arise from the consumption of a single serving of species accumulating Pb. For Cd, the risk increases under repeated consumption of accumulating species during a short period, which is probable as the wild mushroom harvest takes place largely over several weeks during the fall. Moreover, these calculations are simplified because mushrooms are not the sole dietary source of detrimental metals. On the other hand, one should consider the bioavailability of Cd and Pb from investigated mushrooms for humans. Unfortunately, information on species of trace elements in mushrooms, including elements with a potential health risk, has been very limited until now (Kalač 2010). As demonstrated, cooking methods such as boiling or microwaving with water can significantly decrease the content of toxic metals in mushrooms (Svoboda et al. 2002) and moreover, lower their bioaccessibility in the human gastrointestinal tract (Sun et al. 2012). Therefore, the conclusive health risks cannot be fully assessed through investigations of total metal content in the fruiting bodies of mushrooms.

## Conclusions

Some 400 original papers dealing with trace elements in mushroom fruit bodies have been published to date. Several elements or even only one (e.g., Hg or Se) was mostly determined. Recent ICP instruments enable researchers to quantify many elements including those so far very limited in terms of both data on content and knowledge of their biological roles. Furthermore, statistical methods such as PCA and HCA help to reveal new relationships within multielemental files. The adaptation of such tools and methods can be advantageous for the resolution of natural relations dealing with trace metal bioaccumulation/bioexclusion in various mushroom species. This article counts as one of the initial studies set within the multielemental research conception of mushrooms.

## Electronic supplementary material

Below is the link to the electronic supplementary material.

ESM 1 (DOCX 22.7 kb)
