# Supplementary material for: Multielemental analysis of 20 mushroom species growing near a heavily trafficked road in Poland
Source: Environ Sci Pollut Res Int. 2016 May 7;23:16280–95. doi: 10.1007/s11356-016-6760-8 (PMC4975766; doi:10.1007/s11356-016-6760-8)
Supplement: Supplementary file 1 — (DOCX 22.7 kb) [file 11356_2016_6760_MOESM1_ESM.docx]

**Supplementary data to:**

**Biological diversity of *Salix* taxa in remediation of contaminated area**

Mirosław Mleczek^1*^, Paweł Rutkowski^2^, Zygmunt Kaczmarek^3^, Piotr Golinski^1^, Kinga Szentner^1^,

Bogusława Waliszewska^4^, Mariusz Stolarski^5^, Stefan Szczukowski^5^

**Location description**

Table S1. Climatic data

| **Year** | **T** | **TM** | **Tm** | **PP** | **V** | **RA** | **SN** | **TS** | **FG** | **GR** |
| --- | --- | --- | --- | --- | --- | --- | --- | --- | --- | --- |
| 2008 | 10.5 | 14.5 | 5.8 | 614.65 | 12.1 | 193 | 30 | 24 | 66 | 6 |
| 2009 | 10.1 | 14.2 | 4.4 | 579.15 | 10.9 | 192 | 49 | 31 | 93 | 6 |
| 2010 | 8.6 | 12.7 | 2.9 | 757.12 | 10.8 | 168 | 92 | 20 | 86 | 6 |
| 2011 | 10.5 | 15.0 | 4.3 | 580.58 | 11.5 | 162 | 35 | 26 | 85 | 3 |

T Average annual temperature

TM Annual average maximum temperature

Tm Average annual minimum temperature

PP Rain or snow precipitation total annual

V Annual average wind speed

RA Number of days with rain

SN Number of days with snow

TS Number of days with storm

FG Number of foggy days

GR Number of days with hail

**Verification of obtained results**

Results of the experiment were validated on the basis of three certified reference materials, i.e. NIST 1575a (Pine Needles) from the National Institute of Standards and Technology, Gaithersburg and also NCS DC 73349 (Bush branches and leaves) and NCS DC 73350 (Leaves of poplar), both from the China National Analysis Centre for Iron and Steel, Beijing, China, analysed in every tenth measuring set.

Table S2. Comparison of trace element concentration analyses [mg kg^-1^ in dry weight] on the basis of standard curve and after corrections by three certified reference materials, NIST-1575a, NCS DC 73349 and NCS DC 73350.

| NIST-1575a  (Pine Needles) | | | NCS DC 73350  (Leaves of Poplar) | | | | NCS DC 73349  (Bush Branches and Leaves) | | | |  |
| --- | --- | --- | --- | --- | --- | --- | --- | --- | --- | --- | --- |
| Elements | Certified  value | Authors’ results | Recovery (%) | | Certified value | Authors’ results | Recovery (%) | | Certified value | Authors’ results | Recovery  (%) |
| Cd | 0.233±0.004 | 0.241±0.026 | 103 | | 0.32±0.07 | 0.34±0.05 | 106 | | 0.38* | 0.34±0.03 | 89 |
| Co | 0.061±0.002* | 0.053±0.007 | 87 | | 0.42±0.03 | 0.44±0.06 | 105 | | 0.41±0.05 | 0.39±0.11 | 95 |
| Cr | ------------ | ------------ | ------------ | | 0.55±0.07 | 0.52±0.08 | 95 | | 2.6±0.2 | 2.64±0.19 | 102 |
| Cu | 2.8±0.2 | 2.66±0.24 | 95 | | 9.3±1.0 | 9.27±0.83 | 100 | | 6.6±0.8 | 6.51±0.47 | 99 |
| Pb | 0.167±0.015* | 0.172±0.019 | 103 | | 1.5±0.3 | 1.47±0.27 | 98 | | 47±3 | 45.89±4.26 | 98 |
| Zn | 38±2 | 38.14±1.73 | 100 | | 37±3 | 37.14±2.88 | 100 | | 55±4 | 53.92±6.71 | 98 |

* – reference values

**The analytical parameters of the applied method**

Table S3. Experimental conditions of used methods and statistical parameters of the calibration lines

| Element |  | Cd | Co | Cr | Cu | Pb | Zn |
| --- | --- | --- | --- | --- | --- | --- | --- |
| Wavelength | Nm | 228.8 | 240.7 | 357.9 | 324.8 | 217 | 213.9 |
| Slit width | Nm | 0.5 | 0.2 | 0.2 | 0.5 | 1.0 | 1.0 |
| Lamp current | mA | 4 | 7 | 7 | 4 | 9 | 5 |
| Flame type:  Air:Acetylene |  | 13.5 : 2 | 13.5 : 2 | 13.5 : 3.3 | 13.5 : 2 | 13.5 : 2 | 13.5 : 2 |
| Model |  | Quadratic – provides a second order least squares line forced through zero | | | | | |
| Sensitivity | B(x) | 0.16 | 0.06 | 0.04 | 0.03 | 0.02 | 0.06 |
| *LOD* | mg kg^-1^ | 0.01 | 0.1 | 0.06 | 0.03 | 0.17 | 0.02 |
| Minimum concentration | C_min_/ mg kg^-1^ | 0.09 | 0.19 | 0.26 | 1.37 | 0.89 | 1.48 |
| Maximum concentration | C_max_/ mg kg^-1^ | 3.54 | 3.71 | 5.44 | 24.6 | 17.9 | 26.8 |
| Correlation coefficient | r | 0.9989 | 0.9993 | 0.9991 | 0.9996 | 0.9991 | 0.9992 |

**Figure captions**

Fig. S1 Location of research area

Fig. S2 Spatial distribution of the copper content in the soil, taking into account the wind rose [Stuczyński T, Ratowski K, Polak W (2011) Wyznaczenie zasięgu terytorialnego zanieczyszczenia gleb i roślin na obszarach użytkowanych rolniczo na terenie trzech gmin powiatu głogowskiego. Niepubl. (Determination of territorial contamination of soils and plants in areas used for agriculture in the three municipalities of the Głogów district. Unpublished. In Polish only; here generalized)]

Fig. S3 Spatial distribution of lead content in wheat grain, taking into account the wind rose. [Stuczyński T., Ratowski K, Polak W (2011) Wyznaczenie zasięgu terytorialnego zanieczyszczenia gleb i roślin na obszarach użytkowanych rolniczo na terenie trzech gmin powiatu głogowskiego. Niepubl. (Determination of territorial contamination of soils and plants in areas used for agriculture in the three municipalities of the Głogów district. Unpublished. In Polish only; here generalized)]
